# An exploration of the impact of phylogenetic tree structure on NTI and βNTI estimates of community assembly

**DOI:** 10.1038/s41598-024-74696-8

**Published:** 2024-10-08

**Authors:** Madeleine J.S. Gundersen, Olav Vadstein

**Affiliations:** Department of Biotechnology and Food Science, Faculty of Natural Sciences, NTNU - Norwegian University of Science and Technology, Trondheim, Norway

**Keywords:** Community ecology, Ecological modelling, Ecosystem ecology, Microbial ecology

## Abstract

The nearest taxon index (NTI) and beta nearest taxon index (βNTI) are popular metrics used to infer community assembly in a variety of ecosystems. However, the dependence these metrics have on the underlying phylogenetic tree is often overlooked and can potentially lead to erroneous conclusions. In this paper, we investigated how misclassified sequences in the phylogenetic tree and reduction of the metacommunity richness affect estimates of the assembly processes. We estimated assembly processes in two datasets investigating fish-microbiota interactions. We found that non-bacterial misclassified sequences can drastically change the interpretation of assembly processes. NTI consistently increased after misclassified sequences were removed, which changed the ecological interpretation for 82.8% of the communities in one of the datasets. Similarly, βNTI was strongly affected by misclassified sequences in the phylogenetic tree, changing the ecological interpretation for 44.7% (Dataset 1) and 6.3% (Dataset 2) of the community comparisons. Lower metacommunity richness generally resulted in decreased NTI and increased βNTI, but the effect sizes were small and generally did not result in a change in ecological interpretation. Based on these findings, we recommend: (1) constructing and visually inspecting a phylogenetic tree for 16 S rRNA gene amplicon datasets to identify and remove misclassified sequences, and (2) evaluating the robustness of community assembly conclusions by performing analyses with varying metacommunity richness and clearly specifying the metacommunity used for the null model approach.

**Supplementary Information:**

The online version contains supplementary material available at 10.1038/s41598-024-74696-8.

## Introduction

Quantifying community assembly processes is critical for understanding how patterns of community diversity arise. Local communities assemble from the metacommunity (i.e. the regional population pool) through both deterministic and stochastic processes^1^. An important deterministic process is selection, which occurs because populations have different fitness in the ecosystem. Examples of selection are environmental filtering and biotic interactions^2,3^. Stochastic processes, such as ecological drift, lead to unpredictable fluctuations in population abundances^1–3^. Stochastic processes pose an important statistical challenge in quantifying community assembly, as such processes result in the absence of patterns in the dataset^1^.

During the last decade, there has been a significant increase in papers attempting to quantify community assembly processes in bacterial communities^4^. Null model-based approaches have become particularly popular for this purpose. In null model-based approaches, communities are randomly generated from the dataset under study (i.e. null communities), and the observed community is compared to these null communities. The underlying assumption of null models is that significant deviations from the null communities may suggest that deterministic processes have structured community assembly.

Two commonly used metrics within this framework are the Nearest Taxon Index (NTI) and βNTI (Beta Nearest Taxon Index). These null model-based approaches randomise the phylogenetic tree associated with the metacommunity^5^. The NTI measures the degree of phylogenetic- clustering or overdispersion in a single community (i.e. within a sample). Communities with high phylogenetic clustering are interpreted to be deterministically structured (e.g. by environmental filtering), with the underlying assumption that phylogenetically close populations thrive in the same environment^1,6^. With the same assumption, communities with highly dispersed phylogeny (i.e. overdispersion) are assumed to be structured deterministically through processes such as competitive exclusion. In the absence of clustering or overdispersion, it is inferred that stochastic processes play a more prominent role in community structuring.

βNTI measures the phylogenetic similarity between two communities (i.e. two samples). If two communities are more, or less, phylogenetically similar than expected by chance, it is assumed that the communities have been structured by selection. The NTI and βNTI are therefore metrics that use a phylogenetic tree to assess co-occurrence patterns and are often used to infer underlying community assembly processes. It is important to note that while these metrics provide insights into potential assembly processes, they are based on assumptions and are not definitive evidence of the underlying mechanisms. Study design, environmental factors, and other confounding variables can influence the results and the null model results should be carefully considered.

**Fig. 1:**
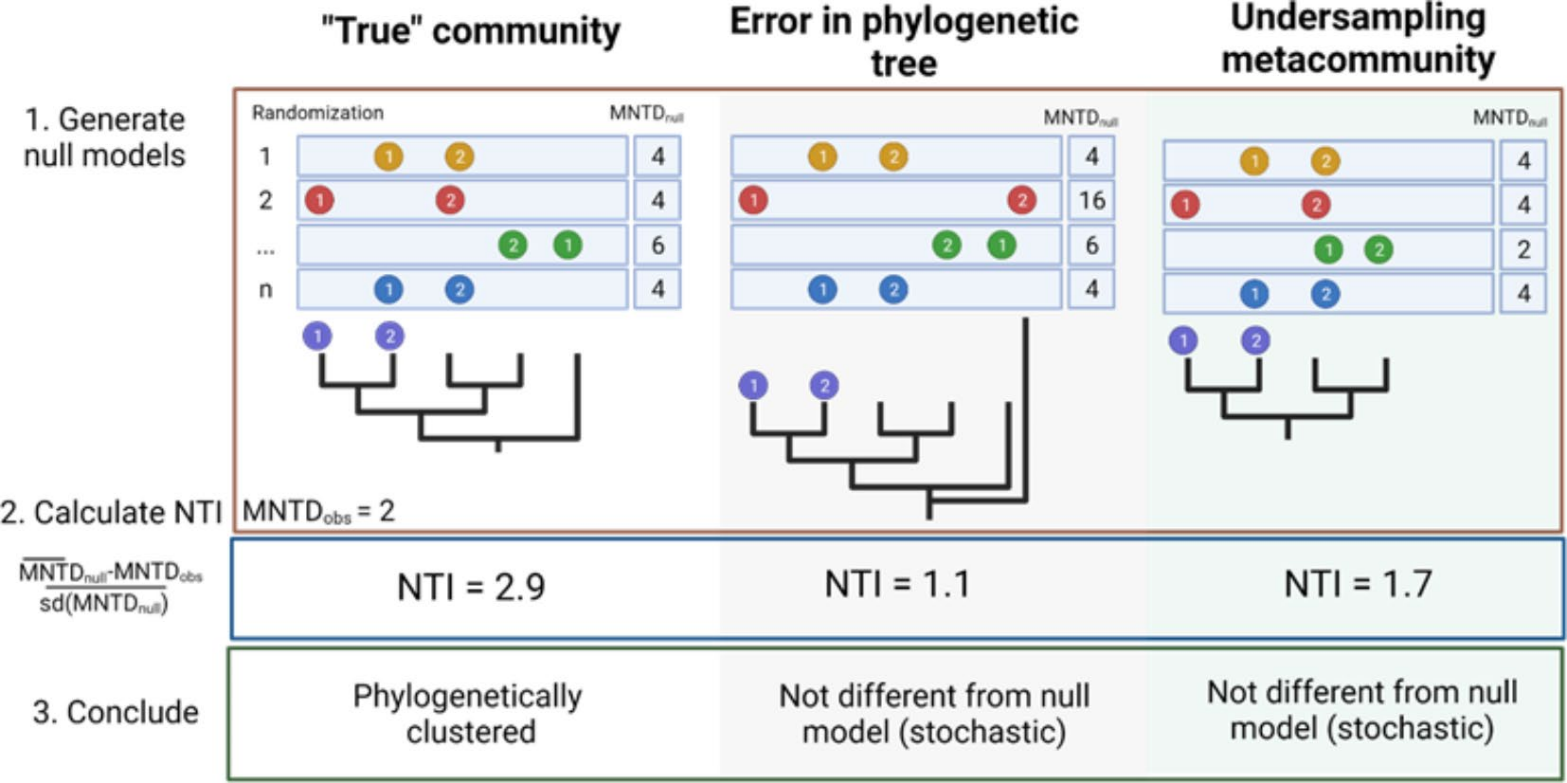
Pitfalls when calculating the nearest taxon unit (NTI). In this simplified example, a community contains two closely related populations (1 and 2) separated on the phylogenetic tree by two branches. Each branch has an arbitrary length of one, resulting in a mean phylogenetic distance (MNTD_observed_) of 2 (1 + 1). Null modelling is performed by randomly shuffling the populations on the tips of the phylogenetic tree, and the MNTD_null_ is calculated for each randomization. The estimated NTI indicates that the community is more phylogenetically clustered than expected by chance (NTI = 2.9 > 2). However, if a sequence not representing the bacterial community is included in the phylogenetic tree, some randomisations will be erroneous and substantially increase the standard deviation of the null models (e.g. randomisation 2 with red dots). This error results in that the community no longer is interpreted as phylogenetically clustered (NTI = 1.1 < 2). Similarly, poor coverage of the metacommunity population pool truncates the phylogenetic tree, and the null model is too similar to the observed community (NTI = 1.7 < 2).

The null model-based metrics rely heavily on the phylogenetic tree of the metacommunity, and several pitfalls can arise when using these metrics. Two such pitfalls are the presence of misclassified sequences in the phylogenetic tree and insufficient coverage of the metacommunity (Fig. 1).

The most common way of analysing bacterial communities is by amplicon sequencing of a variable region of the 16 S rRNA gene. The resulting datasets are large, often containing hundreds to thousands of unique populations and a high proportion of low abundant populations^3^. Datasets based on 16 S rRNA gene amplicon sequencing may contain sequences from organisms other than bacteria, such as archaea and eukaryotic ribosomal sequences. Such non-bacterial sequences can be classified as bacteria during the bioinformatic processing of the sequencing data. Many bacterial ecologists rely on the bioinformatic pipelines to identify the erroneous sequences and filter the dataset to include populations classified as bacteria. Generally, unless the population appears suspicious, its taxonomic classification is not confirmed with additional bioinformatics (e.g. BLAST or RDP classifiers). The presence of such non-bacterial misclassified sequences in the dataset will ultimately result in a phylogenetic tree containing outliers.

A consequence of the presence of non-bacterial sequence outliers is that the phylogenetic tree has more branches than the actual community under study (i.e. bacterial community). The presence of misclassified sequences thus increases the likelihood of communities being evaluated as more overdispersed than the community actually is and might therefore underestimate the degree of deterministic processes (Fig. 1). We hypothesise that such outliers will have a major impact on the NTI and βNTI estimates. To our knowledge, the extent to how non-bacterial outliers affect estimates of the community assembly process has not been investigated.

Furthermore, the NTI and βNTI metrics depend on the metacommunity structure. It is well documented that increased sampling effort increases the metacommunity richness (regional population pool)^3^. Thus, more samples and sample types are expected to increase the metacommunity richness, which in turn affects the size and structure of the phylogenetic tree. However, there are no guidelines to define the cut-off for the metacommunity. It is therefore likely that the mean phylogenetic relationships will change as a function of sampling effort. Fewer samples are expected to cover a smaller proportion of the true metacommunity. The consequence of low coverage is that the randomly generated null communities will, on average, have a higher similarity to the observed community. Such higher similarity will make it less likely to obtain NTI or βNTI that are significantly different from the null model. The importance of metacommunity structure was discussed already by Webb (2000) when he introduced the NTI framework^7^. Still, to our knowledge, there are no guidelines that show how bacterial metacommunity richness impacts the estimates of NTI and βNTI and thus its effect on the estimates needs renewed attention.

This paper investigates the effect of (1) a phylogenetic tree containing non-bacterial misclassified sequences and (2) the metacommunity richness on the results of the NTI and βNTI analyses. We used two datasets examining the bacterial communities associated with fish for this investigation^8,9^. We have previously reported and discussed the community assembly processes for the communities in these datasets^8,9^. During data analysis, we noticed the impact the pitfalls had on the assembly quantifications and address this in the current study. We found that misclassified sequences in the phylogenetic tree had a large impact on the interpretation of the null model results. Our investigation demonstrates the impact the phylogenetic tree has on analysis of assembly and highlight the need for awareness of the underlying assumptions when using phylogenetic based null model approaches. We encourage a more critical evaluation of null model assumptions within the research community.

## Materials and methods

### Bacterial community datasets overview and structure

Two datasets were used to explore the pitfalls related the NTI and βNTI metrics. We have previously reported and discussed the community assembly processes for these datasets^8,9^. Both datasets investigated fish-bacterial community interactions and sampled the bacterial communities associated with fish, rearing water and intake water^8,9^. The following sections briefly give an overview of the structure of the two datasets. A full description of the experimental design and analytical approach used to generate the datasets are presented in Mathisen et al. 2024^9^ (Dataset 1) and Vestrum et al. 2018^8^ (Dataset 2).

In Dataset 1 the bacterial communities in the guts of Atlantic salmon alevins (*Salmo salar*) reared under two different water qualities (*r*- or *K*-selected intake water) were analysed^9^. The bacterial communities were analysed using 16 S rRNA gene amplicon sequencing. An amplicon sequence variant (ASV) table was generated from the sequencing reads using the USEARCH pipeline and the RDP v16 database. The dataset contained 88 samples (8 intake water, 16 rearing water and 64 gut samples) and 3186 ASVs after removal of contaminants.

In Dataset 2 the bacterial communities associated with Atlantic cod (*Gadus mohua*) larvae reared in three different aquaculture systems were analysed^8^. The aquaculture systems were a flow-through system (FTS), a microbially matured water system (MMS) or a recirculating water system (RAS). Throughout the experiment, bacterial community samples were taken of the intake- and rearing water, feed and cod larvae. 16 S rRNA gene amplicon sequencing was used to analyse the communities. The sequencing reads were processed similarly to Dataset 1, but an operational taxonomic unit (OTU) table was generated instead of an ASV Table (97% similarity level). Dataset 2 contained 197 samples (108 cod gut, 48 rearing water, 13 intake water and 28 feed samples) and 3336 OTUs after removal of contaminants. These two datasets were used as input for this exploratory analysis. All data analyses were performed in R^10^ (version 4.2.3), with Datasets 1 and 2 analysed separately. All R scripts are available online at *github.com/madeleine-gundersen/ phytree_NTIbNTI*.

## Phylogenetic tree and outlier identification

To estimate the NTI and βNTI in the two datasets a phylogenetic tree is needed. A phylogenetic tree was constructed by first aligning the 16s rRNA gene sequences using *AlignSeqs*() from the DECIPHER package^11^ (version 2.26.0). From these aligned sequences, a neighbour joining tree was constructed using the *pml()*function from the phangorn package^12^ (version 2.11.1). The tree parameter estimates were further optimized by fitting a generalized time-reversible with Gamma rate variation maximum likelihood tree (GTR + G + I) using the neighbour-joining tree as a starting point. The tree model optimization was performed using *optim.pml()* from phangorn. The trees were rooted to the longest branch length.

**Fig. 2:**
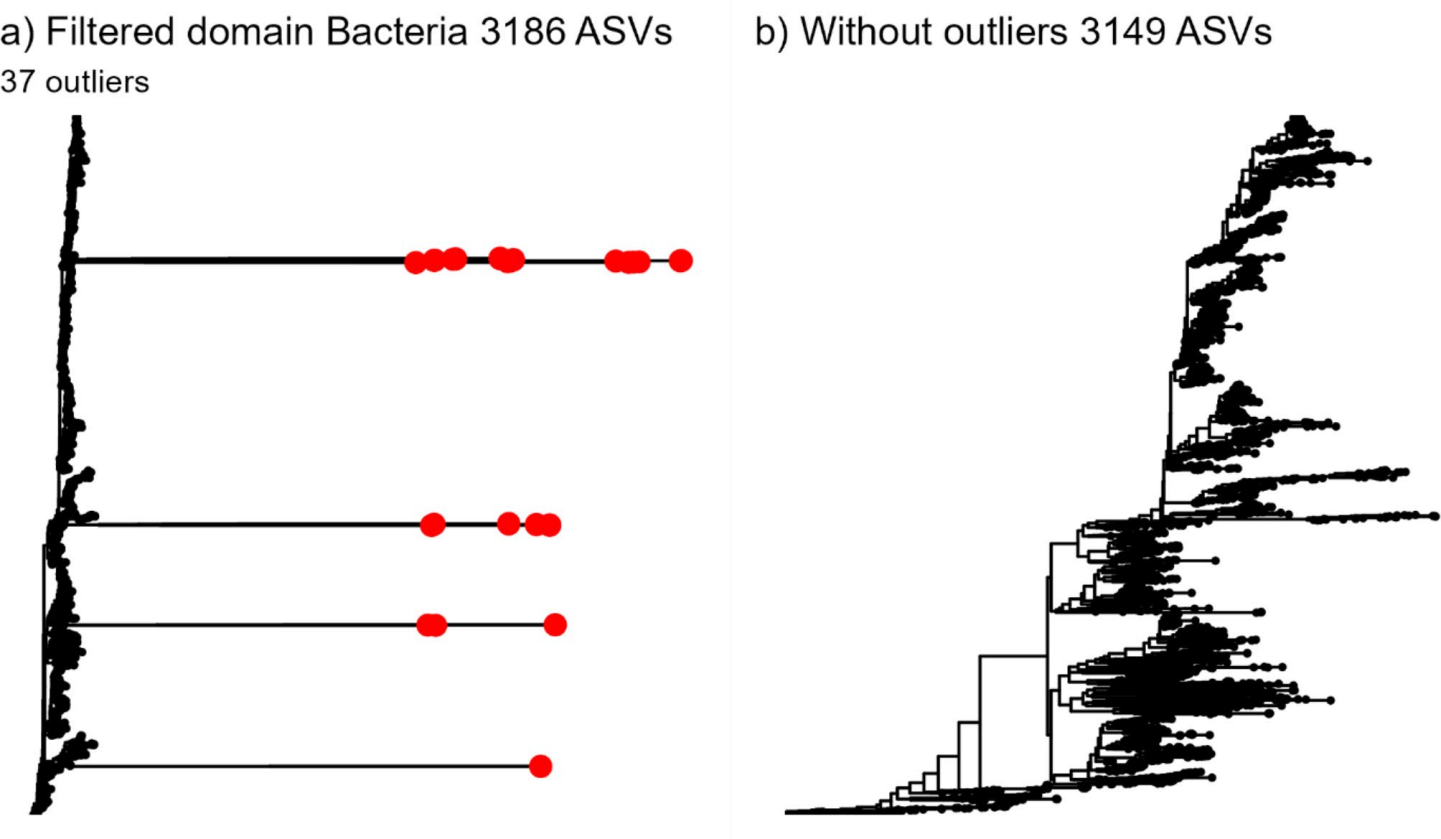
Phylogenetic trees of Dataset 1 (Salmon alevins) in (**a**) the full dataset containing all ASVs classified to belong to the domain Bacteria (3186 ASVs) and (**b**) outlier-free dataset (3149 ASVs). All non-bacterial outliers identified in the full dataset are highlighted in red. The phylogenetic trees show that a few non-bacterial outliers (37) can have an enormous impact on the phylogenetic tree structure.

We were interested in investigating the bacterial communities in Dataset 1 and 2. Thus, we first filtered the datasets to only contain populations classified as belonging to the bacterial domain. We then plotted the phylogenetic trees and observed that some populations were highly divergent from most of the population pool (see Fig. 2a). These divergent populations were suspected to be misclassified sequences. The population ID and sequence information of the suspected misclassified sequences was obtained by extracting the population ID of populations with a branch length above a defined value. This value was set based on the properties of the phylogenetic tree and varied between the dataset investigated. The branch length value was chosen when suspected misclassified sequences were highlighted on the phylogenetic tree (visual inspection). We used functions from the ggtree package^13^(version 3.6.2) to plot the phylogenetic trees and extracted the ID of potential misclassified sequences (for more information see the r-scripts provided in the GitHub repository). These suspected misclassified sequences had been classified to belong to the bacterial domain through the bioinformatical approach used to generate the datasets (see section Bacterial community dataset overview). To further inspect these sequences, we performed a NCBI nucleotide BLAST search for each suspected misclassified sequence separatly^14^. During this inspection, the nucleotide sequence of the suspected misclassified sequence was compared to the NCBI nucleotide database (bacterial, archaeal and eucaryotic database). The search reports a similarity measure to all nucleotides in the database. All suspected misclassified sequences with similarity to taxa other than bacteria were removed from the datasets. We refer to the datasets with misclassified sequence present as «the full» datasets, and those without outliers as «outlier-free» datasets. An R script to identify misclassified sequences is provided in GitHub repository. It should be noted that this method of identifying misclassified sequences is time consuming and tedious. Future projects should develop improved methods to identify the misclassified sequences.

## Bacterial community characteristics

To investigate how the removal of the misclassified sequences affected community characteristics, we investigated the change in sample reads, richness and β-diversity. We determined the difference in richness between the full and the outlier-free datasets using the *ntaxa*() function from the phyloseq package^15^(version 1.41.1). To compare how community composition changed after removing the misclassified sequences, we calculated four similarity indices: Bray-Curtis, Sørensen, Weighted (w) and Unweighted (uw) Unifrac. The similarities were calculated for each sample and used to compare the community composition in the full and outlier-free dataset. The similarity should be 1 if the samples are unaffected by the removal of misclassified sequences. Bray-Curtis quantifies the similarity in community composition and considers differences in relative abundance. The Sørensen similarity quantifies the similarity in the presence-absence of populations^16^. The UniFrac indices incorporate both the composition of the bacterial community and the phylogenetic relationship between populations^17^. The unweighted UniFrac does not incorporate relative abundance, whereas the weighted UniFrac does. The similarities were calculated using *distance*() from phyloseq.

## Phylogenetic signal

The underlying assumption of the NTI and βNTI metrics is that environmental conditions select for or against particular populations. These selective processes should be detected in the dataset as a phylogenetic signal. A phylogenetic signal is detected if populations with close phylogenetic relationships co-occur in the same environmental variables. We used the method provided in Stegen et al. 2013 to test for, and confirm, the presence of phylogenetic signal in the outlier-free datasets before estimating NTI and βNTI^5^ (Supplementary Fig. 1). In that approach, the abundance-weighted mean for environmental variables is first standardised to the maximum phylogenetic distances. This standardised weighted mean is then correlated to all phylogenetic distances. A phylogenetic signal is identified if short phylogenetic distances are statistically significant which indicates that closely related populations thrive in the same environmental conditions. In Dataset 1 we used sample type, fish flask, intake water selection regime and host strain as the environmental variables. In Dataset 2 we used sample type, aquaculture system and rearing tank as the environmental variables. Then, a multivariate Mantel correlogram was calculated based on the standardised weighted mean and phylogenetic distances using the function *mantel.correlog()*from the vegan package^18^ (version 2.6-4). A Mantel statistical test was used to infer statistical significance between the two matrices and was Holm-corrected with 1000 permutations.

## Estimation of NTI and βNTI

To quantify the degree of phylogenetic clustering in a single community we used the NTI metric^7^. To estimate NTI, we first generated 1000 null communities by randomising population labels across the phylogenetic tree tips and calculated the mean nearest taxon distances (MNTD) for each null community. The randomisations were performed using the function *ses.mntd*() from the picante package^19^ (version 1.8.2). The randomisation generates stochastically assembled communities (i.e. null communities). NTI was then calculated as the difference between the mean phylogenetic distance of the null communities (mean MNTD_null_) and the observed phylogenetic distance between co-occurring populations in a community (MNTD_observed_), divided by the standard deviation of the null distribution of MNTD_null_. NTI values greater than zero indicate phylogenetic clustering (i.e. populations are more closely related than expected by chance). Conversely, values less than zero indicate phylogenetic overdispersion (i.e. populations are more distantly related than expected by chance). A two-standard deviation is considered significantly different from the null model, and as such NTI <-2 or NTI > 2 is assumed to reflect deterministic community assembly processes.

To estimate the average phylogenetic distance between two communities rather than within a community, we used βNTI^20^. For each community, 1000 null communities are generated by randomly shuffling the population labels in the phylogenetic tree. For the observed and null communities, the βMNTD (β mean nearest taxon distance) is calculated and a βMNTD_null_ distribution is obtained. βNTI is then calculated as the difference between the observed βMNTD and the mean βMNTD of the null communities, divided by the standard deviation of the null distribution of the βMNTD_null_. We estimated βNTI using the function *qpen*() from the iCAMP package^21^ (version 1.5.12).

A two-standard deviation from the null model is considered significant. Community comparisons with a βNTI > 2 share significantly fewer and more dispersed populations than expected by chance. Such phylogenetic patterns are expected to mainly be driven by heterogeneous selection processes. In contrast, community comparisons with a βNTI<-2 share significantly more populations than expected by chance. These communities are assumed to be structured by homogeneous selection because they are more phylogenetically clustered than expected by chance. Community comparisons with a βNTI between − 2 and 2 are not significantly different form the null model and are assumed to be structured by stochastic processes.

We estimated NTI and βNTI for Dataset 1 and 2 separately for different metacommunity subsets of the datasets. The different subsets were (1) the full dataset, (2) outlier-free dataset, (3) rearing water and fish samples only and (4) fish samples only. For each subset all populations present were regarded as part of the metacommunity and used for the null model randomisations. Subsets 3 and 4 were used to assess how reducing the richness of the metacommunity impacted the estimates. Removal of sample-types from the metacommunity should not be done without a basis in biology or experimental design and was solely performed here as an exploratory approach.

## Results and discussion

### Both datasets had misclassified sequences that impacted community characteristics

In Dataset 1, eukaryotic sequences had been classified as bacteria by the SINTAX command in USEARCH. These errors were not detectable in the taxonomy table as all ASVs were classified to the domain *Bacteria*. However, when inspecting the phylogenetic tree, we identified several ASVs with sequences that were divergent from most other ASV sequences (Fig. 2a). A NCBI nucleotide BLAST search on the ASVs that diverged from the other sequences indicated that these ASVs had high similarity to salmonid fish (Supplementary Table 1). When these 37 ASVs (1.2% of all ASVs) were removed, the phylogenetic tree appeared to represent only bacteria, as the tree was uniform with no obvious visual outliers (Fig. 2b). Similarly, in Dataset 2, some OTUs were misclassified in the bioinformatical pipelines. 42 OTUs (1.3% of all OTUs) were identified as misclassified, and their sequences were examined in a NCBI nucleotide BLAST search. The misclassified sequences had a high similarity to Atlantic cod and several different types of fungi (Supplementary Table 2, Supplementary Fig. 2).

In general, removal of the misclassified sequences affected community metrics that use the phylogenetic tree as input. This was despite the fact that the 37 misclassified sequences in Dataset 1 and the 42 misclassified sequences in Dataset 2 constituted a marginal fraction of the population pool and they were relatively rare in each sample (mean sample abundance around 0.1%, Supplementary Table 3).

To test how removing the misclassified sequences affected the community composition in each sample, we quantified the similarity between the full and outlier-free datasets for each sample. We found that Bray Curtis, Sørensen and weighted UniFrac were largely unaffected by removing the outliers (average similarity > 0.97, Supplementary Table 4, Supplementary Fig. 3). While most samples investigated with weighted UniFrac were unaffected, some occurrences had substantially decreased similarity (e.g. one samples had weighted UniFrac similarity of 0.56). The effect of removing the non-bacterial sequences was drastic for the unweighted UniFrac similarity, with 93.2% and 25.9% of the samples in Dataset 1 and 2, respectively, having a similarity below 0.95 (mean ± SD Dataset 1: 0.37 ± 0.22, Dataset 2: 0.97 ± 0.044). Furthermore, there was a tendency for the similarity to increase more when the sample contained more non-bacterial populations (Supplementary Fig. 3). This reduction in unweighted UniFrac similarity clearly illustrates the enormous effect that errors in the phylogenetic tree can have.

It should be noted that the approach used to identify misclassified sequences should be improved. Here, the phylogenetic trees were inspected visually and sequences with a longer branch-length than most of the sequences were investigated in more detail through a nucleotide BLAST search. This process of visual inspection and sequence investigation was repeated until the tree appeared to be without misclassified outliers. It would be advantageous to develop a universal framework to identify all divergent sequences in one procedure. For example, by developing a method to standardize the branch length and investigating all sequences with a standardized branch length above a specified threshold. We therefore encourage the development of a toolbox to identify misclassified sequences.

In conclusion, investigations of the community properties in Datasets 1 and 2 indicate that non-phylogenetic tree based analytical approaches are unaffected by the presence of some misclassified sequences (Bray-Curtis and Sørensen). However, when the phylogenetic tree is crucial for the analytical framework the presence of misclassified sequences introduce bias, especially when considering presence-absence (unweighted UniFrac).

## Effect of misclassified sequences on interpreted community assembly processes

Both NTI and βNTI are dependent on the phylogenetic tree as this is the foundation for the null model randomisation. Misclassified sequences present in the tree can therefore introduce significant bias in the assembly assessment.

**Fig. 3:**
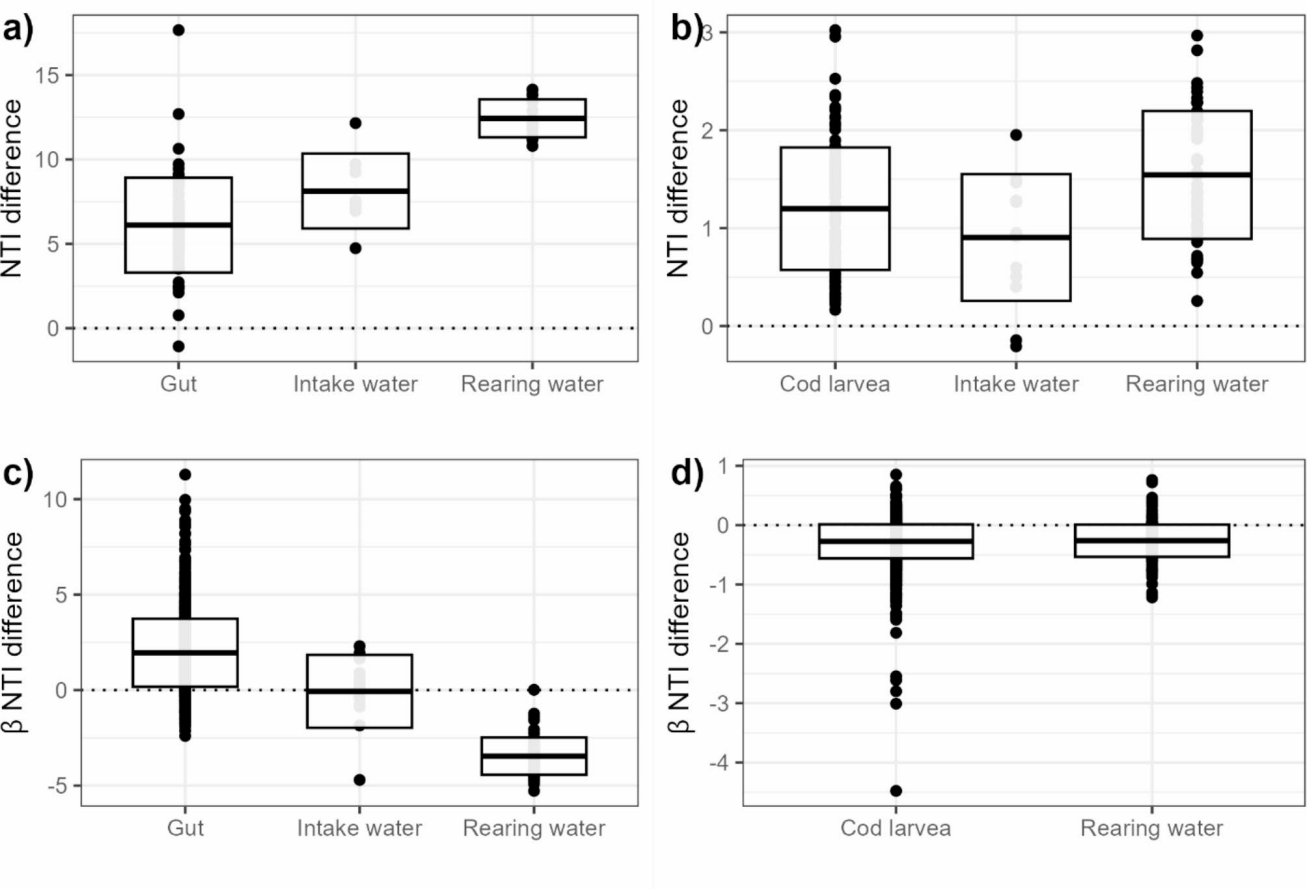
The difference in NTI and βNTI between the full and outlier-free datasets in Dataset 1 (**a**, **c**) and Dataset 2 (**b** and **d**) for each sample type. The box plot shows the mean and standard deviation. The points indicate the NTI in a single sample (**a** and **b**) or the βNTI from a comparison of two samples (**c **and **d**). For Dataset 1, the βNTI was estimated within each sample type (gut, intake and rearing water) and intake water selection regime (r and K selection). For Dataset 2, βNTI was estimated within each sample type (gut and rearing water), sampling day and water treatment regime (FTS, MMS and RAS).

NTI was estimated for each fish, intake and rearing water sample in the datasets with and without the outlier sequences. In general, removal of the non-bacterial outliers increased the NTI (Fig. 3a and b, Supplementary Fig. 4a and c, Supplementary Table 5). For both datasets, the NTI increased for 98.8% of the communities when the misclassified sequences were removed. On average, the NTI increased by 6.1 ± 2.8, 8.1 ± 2.2 and 12.4 ± 1.1 in the salmon gut, intake and rearing water communities, respectively, in Dataset 1 (mean ± SD throughout the results). For the water samples, the conclusion that the communities were phylogenetically clustered remained the same, although the strength of the conclusion was stronger when the misclassified sequences were removed (Table 1, Supplementary Table 5). The changes in NTI after removing the misclassified sequences, led to a different ecological interpretation for 82.8% of the gut communities. Some of the NTI changes were so large that for 9.4% of the gut communities the conclusion regarding community assembly changed from phylogenetically overdispersed to phylogenetically clustered.

For Dataset 2, the NTI did not change as drastically (Table 1, Supplementary Table 5). On average, the NTI increased by 1.3 ± 0.65 and all communities were interpreted as phylogenetically clustered in both the full and outlier-free datasets. However, there was a clear tendency that removing the misclassified sequences consistently resulted in higher NTI. Thus, if the observed MNTD had been closer to the MNTD null model distribution, more sample NTIs might have changed from being interpreted as stochastic to clustered.

**Table 1:** The NTI was calculated in the datasets with and without misclassified outlier sequences. For each sample type, the NTI conclusion was assessed and the number of times (n) the analytical conclusion changed was counted (bold). The percentage of n is given for each sample type (% changed) and each assembly process (% of samples). D = phylogenetically dispersed, C = phylogenetically clustered, S = stochastic.

	Sample Type	% changed	Conclusion full	Conclusion outlier-free	*n*	% of samples
Dataset 1	Salmon gut	82.8	**D**	**C**	**6**	**9.3**
			**D**	**S**	**4**	**6.3**
			**S**	**C**	**43**	**67.2**
			C	C	9	14.1
			S	S	2	3.1
	Rearing water	0	C	C	16	100
	Intake water	0	C	C	8	100
Dataset 2	Cod larvae	0	C	C	108	100
	Rearing water	0	C	C	13	100
	Intake water	0	C	C	48	100

The βNTI was estimated for salmon gut, intake and rearing water in Dataset 1 and for cod larvae and rearing water in Dataset 2. For Dataset 1, most of the estimated βNTI changed when the misclassified sequences were removed from the dataset. For Dataset 2, the βNTI did not change significantly for most of the community comparisons (Fig. 3c and d, Supplementary Fig. 4b and d, Supplementary Table 5). For the water samples, the general trend was that βNTI decreased when the misclassified sequences were removed. The decrease in βNTI led to a change in the interpreted assembly process from stochastic to homogeneous selection for 62.5% of the rearing water and 25.0% of the intake water community comparisons in Dataset (1) Similar changes in the interpreted assembly process were observed for 6.7% of the rearing water community comparisons in Dataset 2 (Table 2). For the fish samples, the general trend was that the βNTI increased in Dataset 1 and decreased in Dataset (2) In Dataset 1, 91.0% of the salmon gut community comparisons had a higher βNTI. These increases resulted in the interpreted assembly processes changing from stochastic to heterogeneous selection dominating the community assembly in 44.0% of the gut community comparisons. In Dataset 2, 78.7% of the cod larvae community comparisons decreased in βNTI, resulting in a change in interpreted assembly process for 6.3% of the community comparisons.

In Dataset 1, the effect of non-bacterial outlier sequences on βNTI estimates was drastic. The change in βNTI resulted in a change in the interpreted assembly processes for 44.7% of the community comparisons. In Dataset 2, the effect of change in βNTI was less pronounced, as only 6.3% of the community comparisons changed with respect to the assembly processes conclusion. These changes in the conclusion regarding the assembly process illustrate that erroneous conclusions can be made if misclassified sequences are present in the phylogenetic tree.

**Table 2:** The βNTI was calculated in the datasets with and without misclassified outlier sequences. For Dataset 1, the βNTI was estimated within each sample type (gut, intake and rearing water) and intake water selection regime (r and K selection). For Dataset 2, βNTI was estimated within each sample type (gut and rearing water), sampling day and water treatment regime (FTS, MMS and RAS). For each sample type, the βNTI conclusion was assessed and the number of times (n) the analysis conclusion changed was counted (bold). The percentage of n is given for each sample type (% changed) and assembly process (% comparisons). HeS = heterogeneous selection, HoS = homogeneous selection, S = stochastic.

	Sample type	% Changed	Conclusion full	Conclusion outlier-free	*n*	% Comparisons
Dataset 1	Salmon gut	44.0	**HeS**	**S**	**8**	**0.8**
			**S**	**HeS**	**428**	**43.1**
			HeS	HeS	13	1.3
			S	S	543	54.7
	Intake water	25.0	**S**	**HoS**	**3**	**25.0**
			HoS	HoS	1	8.3
			S	S	8	66.7
	Rearing water	62.5	**S**	**HoS**	**35**	**62.5**
			HoS	HoS	20	35.7
			S	S	1	1.8
Dataset 2	Cod larvae	6.3	**HoS**	**S**	**2**	**0.5**
			**S**	**HeS**	**2**	**0.5**
			**S**	**HoS**	**23**	**5.3**
			HoS	HoS	220	50.9
			S	S	185	42.8
	Rearing water	6.7	**S**	**HoS**	**3**	**6.7**
			HoS	HoS	32	71.1
			S	S	10	22.2

Furthermore, the shifts observed in both NTI and βNTI, and the resulting inferred assembly processes, underscore the importance of exercising caution when using these tools. While these metrics can provide insights into community assembly, they should not be considered definitive indicators of deterministic or stochastic processes without careful consideration of the study design, potential confounding factors, and the limitations of the methodology. Misinterpretation can occur if statistical results are over-relied upon without contextual biological understanding.

In conclusion, errors in the phylogenetic tree can lead to substantially different interpretations of the underlying ecological mechanisms in community assembly deduced from both NTI and βNTI.

### Effect of metacommunity richness on interpreted community assembly processes

The richness of the metacommunity will impact the structure of the phylogenetic tree, and we therefore investigated the effect of metacommunity richness on NTI and βNTI. In Dataset 1 there were three different sample types: salmon gut, intake- and rearing water. Dataset 2 had four different sample types: cod larvae, feed, intake- and rearing water. As the focus of these experiments was on the bacterial communities of the fish, it is reasonable to imagine that the experiments could have been carried out without sampling the intake or rearing water, or the feed. It is expected that more sample types will result in a larger metacommunity due to the richness-sample relationship^3^, and thus a higher coverage of the true metacommunity. To explore the effect of metacommunity richness, we excluded some sample types and investigated how a metacommunity with lower richness affected the NTI and βNTI estimates.

For both Datasets 1 and 2, we used the outlier-free dataset as the initial metacommunity. The data were then filtered to obtain a metacommunity consisting of only fish and rearing water samples (rearing + fish metacommunity) or only fish samples (fish metacommunity). This filtering was performed as an exploratory measure and should not be performed without a basis in biology or experimental design. In Dataset 1, removal of intake water (9.1% of the samples) and all water samples (27.2% of the samples) from the metacommunity resulted in a loss of ASVs of 20.3% (640 ASVs) and 33.1% (1043 ASVs), respectively (Supplementary Table 6). In Dataset 2, removal of feed and intake water (20.8% of the samples) and feed and all water samples (45.2% of the samples) resulted in a loss of 7.0% (230 OTUs) and 42.8% (1411 OTUs) of the regional OTU pool, respectively (Supplementary Table 6).

**Fig. 4:**
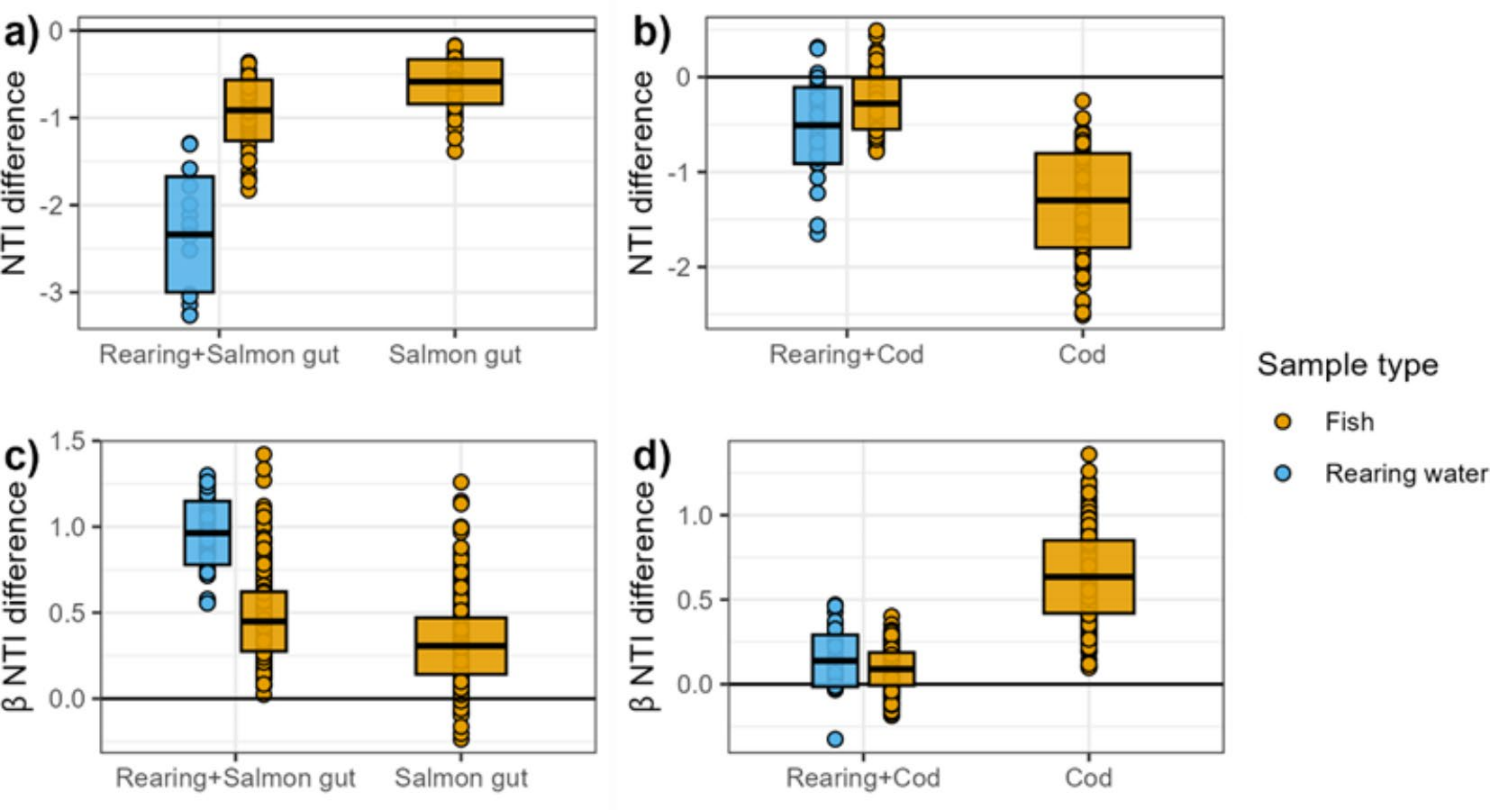
The difference in estimated NTI and βNTI between the whole metacommunity and reduced metacommunity. The difference in estimated NTI for all fish and rearing water samples was calculated as the difference in estimated NTI between the reduced and the original metacommunity in (**a**) Dataset 1 and (**b**) Dataset 2. The difference in estimated βNTI for all fish and rearing water samples was calculated as the difference in estimated βNTI between the reduced and the original metacommunity in (**c**) Dataset 1 and (**d**) Dataset 2. The metacommunity is indicated on the x-axis. The box plot shows the mean change in NTI/ βNTI ± standard deviation.

In general, reducing the metacommunity richness resulted in lower NTI and higher βNTI (Fig. 4, Supplementary Fig. 5). In Dataset 1, the rearing water communities had on average a 2.34 ± 0.66 lower NTI than when all samples were included in the metacommunity (Fig. 4a). Although the NTI was reduced, the conclusion that the rearing water communities were phylogenetically clustered remained. The reduction in NTI was less pronounced in the salmon gut communities, with an average NTI reduction of -0.91 ± 0.35 and − 0.58 ± 0.26 when the intake and all water samples were removed from the metacommunity, respectively. For 6.3% of the gut communities, these NTI reductions resulted in a change in conclusion; the communities were evaluated as stochastically assembled instead of phylogenetically clustered. When the feed and intake water samples were removed from the metacommunity in Dataset 2, NTI decreased on average by -0.51 ± 0.40 in the water communities and − 0.28 ± 0.27 in the cod larvae communities (Fig. 4b). The decrease in the cod larvae communities was higher, with an average of -1.30 ± 0.50 when only the cod larvae samples were present in the metacommunity. Decreasing the metacommunity richness did not result in a change in ecological interpretation of the NTI, and all communities were evaluated as phylogenetically clustered.

In general, a metacommunity with lower richness resulted in a higher βNTI (Fig. 4c and d). When the metacommunity consisted of only rearing water and fish samples the βNTI of the rearing water community comparisons increased on average by 0.96 ± 0.19 in Dataset 1 and 0.14 ± 0.15 in Dataset 2. These increases led to a change in the ecological interpretation of the assembly process for only one community comparison.

When the metacommunity consisted of only the rearing water and salmon gut samples the βNTI increased by an average of 0.45 ± 0.17 for the salmon gut community comparisons in Dataset 1. The increase in βNTI was a bit smaller (0.31 ± 0.16) when the metacommunity only contained the gut samples. Interesting, whereas the changes were relatively small, the change in βNTI resulted in a change in conclusion from stochastic to heterogeneous selection structuring community assembly for 9.2% (rearing + salmon gut metacommunity) and 6.4% (salmon gut metacommunity) of the salmon gut community comparisons. In Dataset 2, the βNTI also increased for the cod larvae community comparisons when the metacommunity was reduced. When only rearing water and cod samples were part of the metacommunity, βNTI increased by 0.090 ± 0.097. When only the cod larvae were part of the metacommunity the βNTI increased as much as 0.64 ± 0.22 for the cod larvae community comparisons. Because the βNTI of the cod larvae comparisons were close to -2 (Supplementary Fig. 5), these relatively small increases in βNTI resulted in a change in the conclusion regarding the assembly processes from heterogeneous selection to stochastic assembly for 2.1% (rearing + cod metacommunity) and 17.1% (cod metacommunity) of the cod larvae community comparisons.

In conclusion, the reduced metacommunity richness resulted in an evaluation of communities as less phylogenetically structured. The NTI decreased and βNTI increased when parts of the metacommunity were removed, and thus the richness of the metacommunity affected the conclusions drawn from these null model-based approaches for both datasets examined. The observations suggest that a more diverse metacommunity increases the likelihood of an ecological community assembly being categorised as deterministic. Consequently, under-sampling increases the likelihood of classification of community assembly as stochastic. However, the changes in βNTI were relatively small, with a maximum difference of about 1.5. Thus, in terms of interpreting ecological processes dominating community assembly, the communities with βNTI close to the null model are most affected. Therefore, we recommend testing the robustness of the conclusions drawn, by filtering out parts of the metacommunity under study and reanalysing the dataset.

## Conclusion

Overall, this exploratory data analysis showed that changes in the phylogenetic tree could affect the magnitude of both NTI and βNTI. We observed that errors in the phylogenetic tree caused by misclassified sequences, had a large impact on the null model-based metrics and a stronger effect size in terms of interpretation of the ecological processes driving community assembly. The effect of a reduction in metacommunity richness had less effect but caused a change in conclusion for a significant part of the communities.

Based on this exploratory investigation, we have two recommendations. First, a phylogenetic tree should always be constructed for 16 S rRNA gene amplicon datasets. It should be standard practice to visualise and inspect this phylogenetic tree, with the aim of identifying whether non-bacterial sequences have been classified as bacteria. Inspecting the tree is a valuable exercise even if no phylogenetic analysis is performed, as it can improve the quality of the dataset. It may also be beneficial to include potentially contaminating host- or ecosystem-related 16/18S rRNA gene sequences in the reference dataset used in bioinformatics pipelines to minimise the inclusion of non-bacterial sequences. Second, if the calculated NTI or βNTI is close to the null model estimates, the analysis should be performed with different metacommunity richness to assess the robustness of the community assembly conclusions. It should also be clearly stated what the metacommunity is based on when describing the analytical approach, as there is a tendency for more comparisons to be classified as deterministic when the metacommunity is more diverse.

In conclusion, this exploratory data analysis highlights some pitfalls of the null model-based approach by emphasising the influence of the phylogenetic tree on the estimation of NTI and βNTI. We stress the importance of constructing accurate phylogenetic trees and taking metacommunity richness into account when using the NTI and βNTI null model framework.

## Electronic supplementary material

Below is the link to the electronic supplementary material.

Supplementary Material 1

## Data Availability

The Illumina sequencing reads are deposited at the European Nucleotide Archive (Dataset 1: ERS17698177-ERS17698264), Dataset 2: accession numbers ERS4778574– ERS4778759). All R-analysis is publicly available at https://github.com/madeleine-gundersen/phytree_NTIbNTI.
