# Supplementary material for: An exploration of the impact of phylogenetic tree structure on NTI and βNTI estimates of community assembly
Source: Sci Rep. 2024 Oct 8;14:23480. doi: 10.1038/s41598-024-74696-8 (PMC11461798; doi:10.1038/s41598-024-74696-8)
Supplement: Supplementary file 1 — Supplementary Material 1 [file 41598_2024_74696_MOESM1_ESM.pdf]

Supplementary figures and tables for:

# An exploration of the impact of phylogenetic tree structure on NTI and βNTI estimates of community assembly

Madeleine J.S. Gundersen, Olav Vadstein

Department of Biotechnology and Food Science

Faculty of Natural Sciences

NTNU - Norwegian University of Science and Technology

Trondheim, Norway

Contact: madeleine.j.s.gundersen@gmail.com

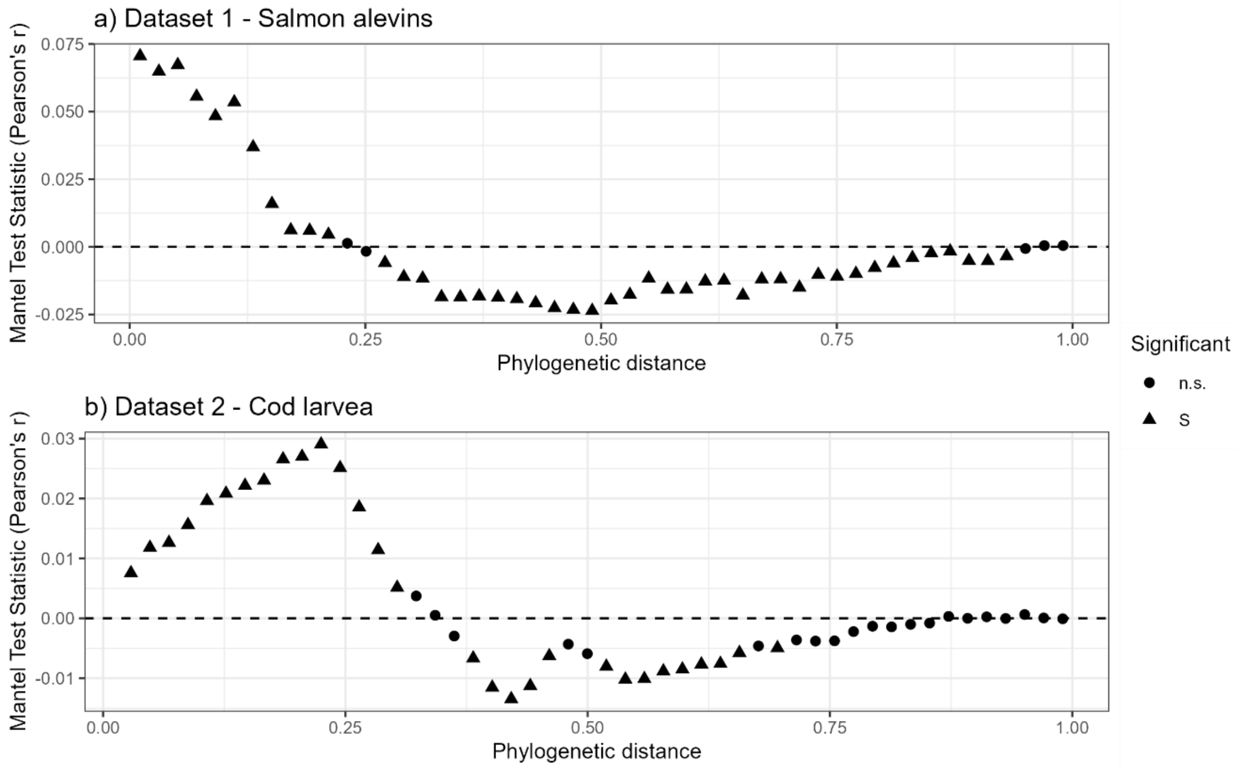


**Supplementary Figure 1:** Phylogenetic Mantel correlogram indicating significant phylogenetic signals across short phylogenetic distances in a) Dataset 1 and b) Dataset 2. Significance indicates whether between ASV/OTU niche is correlated to differences between ASV/OTU phylogenetic differences across a phylogenetic distance (s=significant, p<0.05, n.s. = not significant). The Mantel correlogram indicates that ASVs/OTUs inhabiting the same niches have close phylogenetic distances, and thus there is a phylogenetic signal in the dataset.

**Supplementary Table 1:** The taxonomic identity of the top BLAST matches with highest similarity to the misclassified sequences in Dataset 1. A maximum of five matches and only unique taxonomies are presented for each ASV.

| ASV | Taxonomy |
|---|---|
| **Zotu348** | Eukaryota; Metazoa; Chordata; Craniata; Vertebrata; Euteleostomi; Actinopterygii; Neopterygii; Teleostei; Protacanthopterygii ; Salmoniformes; Salmonidae; Salmoninae; Salmo |
| **Zotu348** | Eukaryota; Metazoa; Chordata; Craniata; Vertebrata; Euteleostomi; Actinopterygii; Neopterygii; Teleostei; Protacanthopterygii ; Salmoniformes; Salmonidae; Coregoninae; Coregonus |
| **Zotu530** | Eukaryota; Metazoa; Chordata; Craniata; Vertebrata; Euteleostomi; Actinopterygii; Neopterygii; Teleostei; Protacanthopterygii ; Salmoniformes; Salmonidae; Salmoninae; Salmo |
| **Zotu592** | Eukaryota; Metazoa; Chordata; Craniata; Vertebrata; Euteleostomi; Actinopterygii; Neopterygii; Teleostei; Protacanthopterygii ; Salmoniformes; Salmonidae; Coregoninae; Coregonus |
| **Zotu592** | Eukaryota; Metazoa; Chordata; Craniata; Vertebrata; Euteleostomi; Actinopterygii; Neopterygii; Teleostei; Protacanthopterygii ; Salmoniformes; Salmonidae; Salmoninae; Salmo |
| **Zotu614** | Eukaryota; Metazoa; Chordata; Craniata; Vertebrata; Euteleostomi; Actinopterygii; Neopterygii; Teleostei; Protacanthopterygii ; Salmoniformes; Salmonidae; Salmoninae; Salmo |
| **Zotu614** | Eukaryota; Metazoa; Chordata; Craniata; Vertebrata; Euteleostomi; Actinopterygii; Neopterygii; Teleostei; Protacanthopterygii ; Salmoniformes; Salmonidae; Coregoninae; Coregonus |
| **Zotu729** | Eukaryota; Metazoa; Chordata; Craniata; Vertebrata; Euteleostomi; Actinopterygii; Neopterygii; Teleostei; Protacanthopterygii ; Salmoniformes; Salmonidae; Salmoninae; Salmo |
| **Zotu729** | Eukaryota; Metazoa; Chordata; Craniata; Vertebrata; Euteleostomi; Actinopterygii; Neopterygii; Teleostei; Neoteleostei; Acant homorphata; Eupercaria; Perciformes; Notothenioidei; Channichthyidae; Pseudochaenichthys |
| **Zotu729** | Eukaryota; Metazoa; Chordata; Craniata; Vertebrata; Euteleostomi; Actinopterygii; Neopterygii; Teleostei; Neoteleostei; Acant homorphata; Eupercaria; Perciformes; Notothenioidei; Bovichtidae; Cottoperca |
| **Zotu729** | Eukaryota; Metazoa; Chordata; Craniata; Vertebrata; Euteleostomi; Actinopterygii; Neopterygii; Teleostei; Protacanthopterygii ; Argentinidae; Argentina |
| **Zotu910** | Eukaryota; Metazoa; Chordata; Craniata; Vertebrata; Euteleostomi; Actinopterygii; Neopterygii; Teleostei; Protacanthopterygii ; Salmoniformes; Salmonidae; Salmoninae; Salmo |
| **Zotu910** | Eukaryota; Metazoa; Chordata; Craniata; Vertebrata; Euteleostomi; Actinopterygii; Neopterygii; Teleostei; Protacanthopterygii ; Salmoniformes; Salmonidae; Coregoninae; Coregonus |
| **Zotu910** | Eukaryota; Metazoa; Chordata; Craniata; Vertebrata; Euteleostomi; Actinopterygii; Neopterygii; Teleostei; Protacanthopterygii ; Salmoniformes; Salmonidae; Salmoninae; Salvelinus |
| **Zotu969** | Eukaryota; Metazoa; Chordata; Craniata; Vertebrata; Euteleostomi; Actinopterygii; Neopterygii; Teleostei; Protacanthopterygii ; Salmoniformes; Salmonidae; Salmoninae; Salmo |
| **Zotu969** | Eukaryota; Metazoa; Chordata; Craniata; Vertebrata; Euteleostomi; Actinopterygii; Neopterygii; Teleostei; Protacanthopterygii ; Salmoniformes; Salmonidae; Coregoninae; Coregonus |
| **Zotu1008** | Eukaryota; Metazoa; Chordata; Craniata; Vertebrata; Euteleostomi; Actinopterygii; Neopterygii; Teleostei; Protacanthopterygii ; Salmoniformes; Salmonidae; Salmoninae; Salmo |
| **Zotu1008** | Eukaryota; Metazoa; Chordata; Craniata; Vertebrata; Euteleostomi; Actinopterygii; Neopterygii; Teleostei; Protacanthopterygii ; Salmoniformes; Salmonidae; Coregoninae; Coregonus |
| **Zotu1015** | Eukaryota; Metazoa; Chordata; Craniata; Vertebrata; Euteleostomi; Actinopterygii; Neopterygii; Teleostei; Protacanthopterygii ; Salmoniformes; Salmonidae; Coregoninae; Coregonus |
| **Zotu1015** | Eukaryota; Metazoa; Chordata; Craniata; Vertebrata; Euteleostomi; Actinopterygii; Neopterygii; Teleostei; Protacanthopterygii ; Salmoniformes; Salmonidae; Salmoninae; Salmo |
| **Zotu1020** | Eukaryota; Metazoa; Chordata; Craniata; Vertebrata; Euteleostomi; Actinopterygii; Neopterygii; Teleostei; Protacanthopterygii ; Salmoniformes; Salmonidae; Salmoninae; Salmo |
| **Zotu1096** | Eukaryota; Metazoa; Chordata; Craniata; Vertebrata; Euteleostomi; Actinopterygii; Neopterygii; Teleostei; Protacanthopterygii ; Salmoniformes; Salmonidae; Salmoninae; Salmo |
| **Zotu1177** | Eukaryota; Metazoa; Chordata; Craniata; Vertebrata; Euteleostomi; Actinopterygii; Neopterygii; Teleostei; Protacanthopterygii ; Salmoniformes; Salmonidae; Salmoninae; Salmo |
| **Zotu1177** | Eukaryota; Metazoa; Chordata; Craniata; Vertebrata; Euteleostomi; Actinopterygii; Neopterygii; Teleostei; Protacanthopterygii ; Salmoniformes; Salmonidae; Coregoninae; Coregonus |
| **Zotu1266** | Eukaryota; Metazoa; Chordata; Craniata; Vertebrata; Euteleostomi; Actinopterygii; Neopterygii; Teleostei; Protacanthopterygii ; Salmoniformes; Salmonidae; Salmoninae; Salmo |
| **Zotu1266** | Eukaryota; Metazoa; Chordata; Craniata; Vertebrata; Euteleostomi; Actinopterygii; Neopterygii; Teleostei; Protacanthopterygii ; Salmoniformes; Salmonidae; Salmoninae; Oncorhynchus |
| **Zotu1334** | Eukaryota; Metazoa; Chordata; Craniata; Vertebrata; Euteleostomi; Actinopterygii; Neopterygii; Teleostei; Protacanthopterygii ; Salmoniformes; Salmonidae; Salmoninae; Salmo |
| **Zotu1349** | Eukaryota; Metazoa; Chordata; Craniata; Vertebrata; Euteleostomi; Actinopterygii; Neopterygii; Teleostei; Protacanthopterygii ; Salmoniformes; Salmonidae; Salmoninae; Salmo |
| **Zotu1349** | Eukaryota; Metazoa; Chordata; Craniata; Vertebrata; Euteleostomi; Actinopterygii; Neopterygii; Teleostei; Protacanthopterygii ; Salmoniformes; Salmonidae; Salmoninae; Oncorhynchus |

| | |
|---|---|
| **Zotu1404** | Eukaryota; Metazoa; Chordata; Craniata; Vertebrata; Euteleostomi; Actinopterygii; Neopterygii; Teleostei; Protacanthopterygii ; Salmoniformes; Salmonidae; Salmoninae; Salmo |
| **Zotu1404** | Eukaryota; Metazoa; Chordata; Craniata; Vertebrata; Euteleostomi; Actinopterygii; Neopterygii; Teleostei; Protacanthopterygii ; Salmoniformes; Salmonidae; Coregoninae; Coregonus |
| **Zotu1404** | Eukaryota; Metazoa; Chordata; Craniata; Vertebrata; Euteleostomi; Actinopterygii; Neopterygii; Teleostei; Protacanthopterygii ; Salmoniformes; Salmonidae; Salmoninae; Oncorhynchus |
| **Zotu1462** | Eukaryota; Metazoa; Chordata; Craniata; Vertebrata; Euteleostomi; Actinopterygii; Neopterygii; Teleostei; Protacanthopterygii ; Salmoniformes; Salmonidae; Salmoninae; Salmo |
| **Zotu1654** | Eukaryota; Metazoa; Chordata; Craniata; Vertebrata; Euteleostomi; Actinopterygii; Neopterygii; Teleostei; Protacanthopterygii ; Salmoniformes; Salmonidae; Salmoninae; Salmo |
| **Zotu1654** | Eukaryota; Metazoa; Chordata; Craniata; Vertebrata; Euteleostomi; Actinopterygii; Neopterygii; Teleostei; Protacanthopterygii ; Salmoniformes; Salmonidae; Salmoninae; Salvelinus |
| **Zotu1742** | Eukaryota; Metazoa; Chordata; Craniata; Vertebrata; Euteleostomi; Actinopterygii; Neopterygii; Teleostei; Protacanthopterygii ; Salmoniformes; Salmonidae; Salmoninae; Salmo |
| **Zotu1742** | Eukaryota; Metazoa; Chordata; Craniata; Vertebrata; Euteleostomi; Actinopterygii; Neopterygii; Teleostei; Protacanthopterygii ; Salmoniformes; Salmonidae; Coregoninae; Coregonus |
| **Zotu1751** | Eukaryota; Metazoa; Chordata; Craniata; Vertebrata; Euteleostomi; Actinopterygii; Neopterygii; Teleostei; Protacanthopterygii ; Salmoniformes; Salmonidae; Salmoninae; Salmo |
| **Zotu1751** | Eukaryota; Metazoa; Chordata; Craniata; Vertebrata; Euteleostomi; Actinopterygii; Neopterygii; Teleostei; Protacanthopterygii ; Salmoniformes; Salmonidae; Coregoninae; Coregonus |
| **Zotu1752** | Eukaryota; Metazoa; Chordata; Craniata; Vertebrata; Euteleostomi; Actinopterygii; Neopterygii; Teleostei; Protacanthopterygii ; Salmoniformes; Salmonidae; Salmoninae; Salmo |
| **Zotu1752** | Eukaryota; Metazoa; Chordata; Craniata; Vertebrata; Euteleostomi; Actinopterygii; Neopterygii; Teleostei; Protacanthopterygii ; Salmoniformes; Salmonidae; Coregoninae; Coregonus |
| **Zotu1766** | Eukaryota; Metazoa; Chordata; Craniata; Vertebrata; Euteleostomi; Actinopterygii; Neopterygii; Teleostei; Protacanthopterygii ; Salmoniformes; Salmonidae; Salmoninae; Salmo |
| **Zotu1766** | Eukaryota; Metazoa; Chordata; Craniata; Vertebrata; Euteleostomi; Actinopterygii; Neopterygii; Teleostei; Protacanthopterygii ; Salmoniformes; Salmonidae; Salmoninae; Salvelinus |
| **Zotu1766** | Eukaryota; Metazoa; Chordata; Craniata; Vertebrata; Euteleostomi; Actinopterygii; Neopterygii; Teleostei; Protacanthopterygii ; Salmoniformes; Salmonidae; Coregoninae; Coregonus |
| **Zotu1801** | Eukaryota; Metazoa; Chordata; Craniata; Vertebrata; Euteleostomi; Actinopterygii; Neopterygii; Teleostei; Protacanthopterygii ; Salmoniformes; Salmonidae; Salmoninae; Salmo |
| **Zotu1801** | Eukaryota; Metazoa; Chordata; Craniata; Vertebrata; Euteleostomi; Actinopterygii; Neopterygii; Teleostei; Protacanthopterygii ; Salmoniformes; Salmonidae; Salmoninae; Oncorhynchus |
| **Zotu1824** | Eukaryota; Metazoa; Chordata; Craniata; Vertebrata; Euteleostomi; Actinopterygii; Neopterygii; Teleostei; Protacanthopterygii ; Salmoniformes; Salmonidae; Salmoninae; Salmo |
| **Zotu1824** | Eukaryota; Metazoa; Chordata; Craniata; Vertebrata; Euteleostomi; Actinopterygii; Neopterygii; Teleostei; Protacanthopterygii ; Salmoniformes; Salmonidae; Salmoninae; Oncorhynchus |
| **Zotu1863** | Eukaryota; Metazoa; Chordata; Craniata; Vertebrata; Euteleostomi; Actinopterygii; Neopterygii; Teleostei; Protacanthopterygii ; Salmoniformes; Salmonidae; Salmoninae; Salmo |
| **Zotu1863** | Eukaryota; Metazoa; Chordata; Craniata; Vertebrata; Euteleostomi; Actinopterygii; Neopterygii; Teleostei; Neoteleostei; Acant homorphata; Eupercaria; Perciformes; Notothenioidei; Channichthyidae; Pseudochaenichthys |
| **Zotu1863** | Eukaryota; Metazoa; Chordata; Craniata; Vertebrata; Euteleostomi; Actinopterygii; Neopterygii; Teleostei; Neoteleostei; Acant homorphata; Carangaria; Pleuronectiformes; Pleuronectoidei; Pleuronectidae; Pleuronectes |
| **Zotu1863** | Eukaryota; Metazoa; Chordata; Craniata; Vertebrata; Euteleostomi; Actinopterygii; Neopterygii; Teleostei; Neoteleostei; Acant homorphata; Eupercaria; Perciformes; Notothenioidei; Bovichtidae; Cottoperca |
| **Zotu1896** | Eukaryota; Metazoa; Chordata; Craniata; Vertebrata; Euteleostomi; Actinopterygii; Neopterygii; Teleostei; Protacanthopterygii ; Salmoniformes; Salmonidae; Salmoninae; Salmo |
| **Zotu1896** | Eukaryota; Metazoa; Chordata; Craniata; Vertebrata; Euteleostomi; Actinopterygii; Neopterygii; Teleostei; Protacanthopterygii ; Salmoniformes; Salmonidae; Coregoninae; Coregonus |
| **Zotu2052** | Eukaryota; Metazoa; Chordata; Craniata; Vertebrata; Euteleostomi; Actinopterygii; Neopterygii; Teleostei; Protacanthopterygii ; Salmoniformes; Salmonidae; Salmoninae; Salmo |
| **Zotu2052** | Eukaryota; Metazoa; Chordata; Craniata; Vertebrata; Euteleostomi; Actinopterygii; Neopterygii; Teleostei; Protacanthopterygii ; Salmoniformes; Salmonidae; Coregoninae; Coregonus |
| **Zotu2052** | Eukaryota; Metazoa; Chordata; Craniata; Vertebrata; Euteleostomi; Actinopterygii; Neopterygii; Teleostei; Ostariophysi; Cyprin iformes; Danionidae; Danioninae; Danio |
| **Zotu2361** | Eukaryota; Metazoa; Chordata; Craniata; Vertebrata; Euteleostomi; Actinopterygii; Neopterygii; Teleostei; Protacanthopterygii ; Salmoniformes; Salmonidae; Salmoninae; Salmo |
| **Zotu2361** | Eukaryota; Metazoa; Chordata; Craniata; Vertebrata; Euteleostomi; Actinopterygii; Neopterygii; Teleostei; Protacanthopterygii ; Salmoniformes; Salmonidae; Coregoninae; Coregonus |
| **Zotu2446** | Eukaryota; Metazoa; Chordata; Craniata; Vertebrata; Euteleostomi; Actinopterygii; Neopterygii; Teleostei; Protacanthopterygii ; Salmoniformes; Salmonidae; Salmoninae; Salmo |
| **Zotu2448** | Eukaryota; Metazoa; Chordata; Craniata; Vertebrata; Euteleostomi; Actinopterygii; Neopterygii; Teleostei; Protacanthopterygii ; Salmoniformes; Salmonidae; Salmoninae; Salmo |

| | |
|---|---|
| **Zotu2448** | Eukaryota; Metazoa; Chordata; Craniata; Vertebrata; Euteleostomi; Actinopterygii; Neopterygii; Teleostei; Protacanthopterygii ; Salmoniformes; Salmonidae; Salmoninae; Oncorhynchus |
| **Zotu2765** | Bacteria; Pseudomonadota; Gammaproteobacteria; Pseudomonadales; Pseudomonadaceae; Pseudomonas<br>- Assumed to be wrongly classified due to highly diverging phylogenetic distance |
| **Zotu2769** | Eukaryota; Metazoa; Chordata; Craniata; Vertebrata; Euteleostomi; Actinopterygii; Neopterygii; Teleostei; Protacanthopterygii ; Salmoniformes; Salmonidae; Salmoninae; Salmo |
| **Zotu2769** | Eukaryota; Metazoa; Chordata; Craniata; Vertebrata; Euteleostomi; Actinopterygii; Neopterygii; Teleostei; Protacanthopterygii ; Salmoniformes; Salmonidae; Coregoninae; Coregonus |
| **Zotu2821** | Eukaryota; Metazoa; Chordata; Craniata; Vertebrata; Euteleostomi; Actinopterygii; Neopterygii; Teleostei; Protacanthopterygii ; Salmoniformes; Salmonidae; Salmoninae; Salmo |
| **Zotu2821** | Eukaryota; Metazoa; Chordata; Craniata; Vertebrata; Euteleostomi; Actinopterygii; Neopterygii; Teleostei; Protacanthopterygii ; Salmoniformes; Salmonidae; Coregoninae; Coregonus |
| **Zotu2935** | Eukaryota; Metazoa; Chordata; Craniata; Vertebrata; Euteleostomi; Actinopterygii; Neopterygii; Teleostei; Protacanthopterygii ; Salmoniformes; Salmonidae; Salmoninae; Salmo |
| **Zotu3167** | Eukaryota; Metazoa; Chordata; Craniata; Vertebrata; Euteleostomi; Actinopterygii; Neopterygii; Teleostei; Protacanthopterygii ; Salmoniformes; Salmonidae; Salmoninae; Salmo |
| **Zotu3250** | Eukaryota; Metazoa; Chordata; Craniata; Vertebrata; Euteleostomi; Actinopterygii; Neopterygii; Teleostei; Protacanthopterygii ; Salmoniformes; Salmonidae; Salmoninae; Salmo |
| **Zotu3250** | Eukaryota; Metazoa; Chordata; Craniata; Vertebrata; Euteleostomi; Actinopterygii; Neopterygii; Teleostei; Protacanthopterygii ; Salmoniformes; Salmonidae; Coregoninae; Coregonus |
| **Zotu3285** | Bacteria; Pseudomonadota; Betaproteobacteria; Burkholderiales; Oxalobacteraceae; Undibacterium<br>- Assumed to be wrongly classified due to highly diverging phylogenetic distance |
| **Zotu3285** | Bacteria; environmental samples |

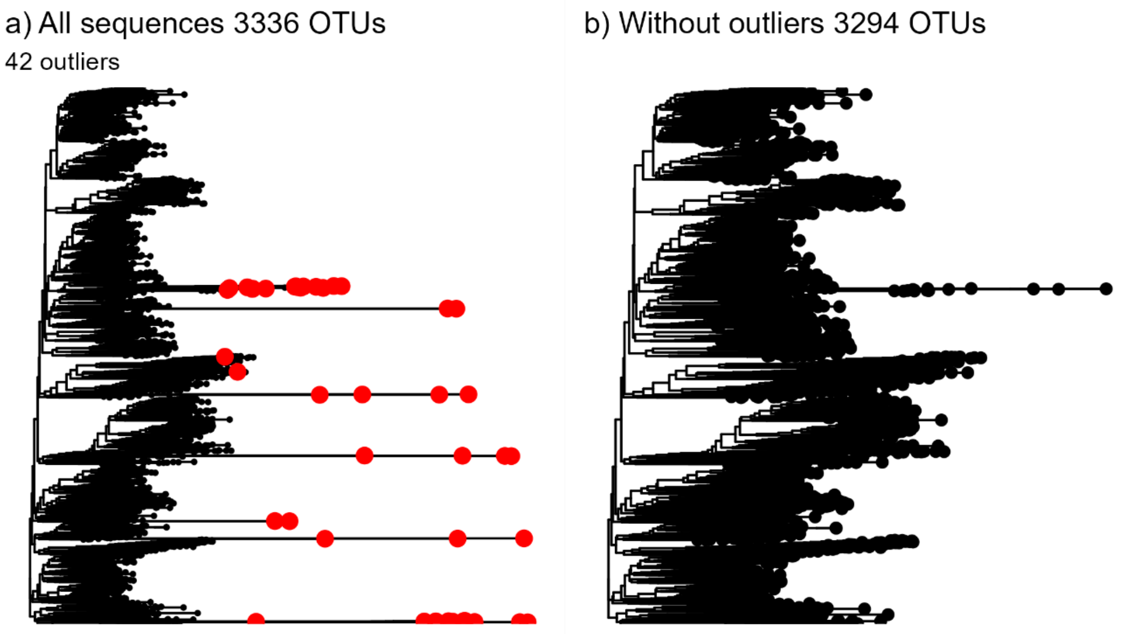


**Supplementary Figure 2:** Phylogenetic trees of Dataset 2 (cod larvae) with a) all OTUs (3336 OTUs) and b) without misclassified outlier sequences (3294 OTUs).

**Supplementary Table 2:** The taxonomic identity of the top BLAST matches with highest similarity to the misclassified outlier sequences in Dataset 2. A maximum of five matches and only unique taxonomies are presented for each ASV.

| OTU | Taxonomy |
|---|---|
| **otu228** | Eukaryota; Fungi; Dikarya; Ascomycota; Pezizomycotina; Dothideomycetes; Pleosporomycetidae; Pleosporales; Delitschiacea e; Delitschia |
| **otu228** | Eukaryota; Fungi; Dikarya; Ascomycota; Pezizomycotina; Dothideomycetes; Dothideomycetes incertae sedis; Zopfiaceae; Zop fia |
| **otu228** | Eukaryota; Fungi; Dikarya; Ascomycota; Pezizomycotina; Dothideomycetes; Pleosporomycetidae; Mytilinidiales; Mytilinidiace ae; Lophium |
| **otu473** | Eukaryota; Sar; Stramenopiles; Bigyra; Opalozoa; Bicosoecida; Caecitellus |
| **otu1022** | no BLAST matches |
| **otu1245** | no BLAST matches |
| **otu1264** | Eukaryota; Fungi; Dikarya; Ascomycota; Pezizomycotina; Sordariomycetes; Hypocreomycetidae; Hypocreales; Nectriaceae; Fu sarium; Fusarium oxysporum species complex |
| **otu1264** | Eukaryota; Fungi; Dikarya; Ascomycota; Pezizomycotina; Leotiomycetes; Helotiales; Dermateaceae; Phlyctema |
| **otu1264** | Eukaryota; Fungi; Dikarya; Ascomycota; Pezizomycotina; Leotiomycetes; Erysiphales; Erysiphaceae; Podosphaera |
| **otu1264** | Eukaryota; Fungi; Dikarya; Ascomycota; Pezizomycotina; Sordariomycetes; Hypocreomycetidae; Hypocreales; Sarocladiaceae; Sarocladium |
| **otu1264** | Eukaryota; Fungi; Dikarya; Ascomycota; Pezizomycotina; Sordariomycetes; Hypocreomycetidae; Hypocreales; Nectriaceae; Fu sarium |
| **otu1421** | Eukaryota; Sar; Stramenopiles; Bigyra; Opalozoa; Bicosoecida; Caecitellus |
| **otu1639** | Eukaryota; Sar; Stramenopiles; Bigyra; Opalozoa; Bicosoecida; Bicosoecida incertae sedis; Bilabrum |
| **otu1639** | Eukaryota; environmental samples |
| **otu1837** | Eukaryota; Sar; Stramenopiles; Bigyra; Opalozoa; Bicosoecida; Caecitellus |
| **otu1837** | Eukaryota; environmental samples |
| **otu1887** | Eukaryota; Metazoa; Chordata; Craniata; Vertebrata; Euteleostomi; Actinopterygii; Neopterygii; Teleostei; Neoteleostei; Acan thomorphata; Zeiogadaria; Gadariae; Gadiformes; Gadoidei; Gadidae; Gadus |
| **otu1945** | Eukaryota; environmental samples |
| **otu1945** | Eukaryota; Sar; Stramenopiles; Bigyra; Opalozoa; Nanomonadea; Incisomonas |
| **otu2101** | Eukaryota; Fungi; Dikarya; Ascomycota; Pezizomycotina; Dothideomycetes; Pleosporomycetidae; Pleosporales; Pleosporinea e; Pleosporaceae; Alternaria; Alternaria sect. Infectoriae |
| **otu2101** | Eukaryota; Fungi; Dikarya; Ascomycota; Pezizomycotina; Dothideomycetes; Pleosporomycetidae; Pleosporales; Pleosporinea e; Pleosporaceae; Alternaria; Alternaria sect. Porri |
| **otu2285** | Eukaryota; Metazoa; Chordata; Craniata; Vertebrata; Euteleostomi; Actinopterygii; Neopterygii; Teleostei; Neoteleostei; Acan thomorphata; Zeiogadaria; Gadariae; Gadiformes; Gadoidei; Gadidae; Gadus |
| **otu2312** | Eukaryota; Fungi |
| **otu2312** | Eukaryota; Fungi; Dikarya; Ascomycota; Pezizomycotina; Dothideomycetes; Pleosporomycetidae; Pleosporales; Pleosporinea e; Didymellaceae; Phoma |
| **otu2312** | Eukaryota; Fungi; Dikarya; Ascomycota; Pezizomycotina; Dothideomycetes; Pleosporomycetidae; Pleosporales; Pleosporinea e; Didymellaceae; Ascochyta |
| **otu2365** | Eukaryota; Sar; Stramenopiles; environmental samples |
| **otu2365** | Eukaryota |
| **otu2365** | Eukaryota; Sar; Stramenopiles; Bigyra; Labyrinthulomycetes; Thraustochytrida; Thraustochytriaceae |
| **otu2365** | Eukaryota; environmental samples |
| **otu2487** | Eukaryota; Metazoa; Chordata; Craniata; Vertebrata; Euteleostomi; Actinopterygii; Neopterygii; Teleostei; Neoteleostei; Acan thomorphata; Zeiogadaria; Gadariae; Gadiformes; Gadoidei; Gadidae; Gadus |
| **otu2487** | Eukaryota; Metazoa; Chordata; Craniata; Vertebrata; Euteleostomi; Actinopterygii; Chondrostei; Acipenseriformes; Acipenser idae; Acipenser |

| | |
|---|---|
| **otu2541** | Eukaryota; Metazoa; Chordata; Craniata; Vertebrata; Euteleostomi; Actinopterygii; Neopterygii; Teleostei; Neoteleostei; Acanthomorphata; Zeiogadaria; Gadariae; Gadiformes; Gadoidei; Gadidae; Gadus |
| **otu2704** | Eukaryota; Metazoa; Chordata; Craniata; Vertebrata; Euteleostomi; Actinopterygii; Neopterygii; Teleostei; Neoteleostei; Acanthomorphata; Zeiogadaria; Gadariae; Gadiformes; Gadoidei; Gadidae; Gadus |
| **otu3087** | Eukaryota; Metazoa; Chordata; Craniata; Vertebrata; Euteleostomi; Actinopterygii; Neopterygii; Teleostei; Neoteleostei; Acanthomorphata; Zeiogadaria; Gadariae; Gadiformes; Gadoidei; Gadidae; Gadus |
| **otu3120** | Bacteria; environmental samples |
| **otu3120** | unclassified sequences; environmental samples |
| **otu3120** | Archaea; environmental samples |
| **otu3159** | Eukaryota; Fungi; Dikarya; Ascomycota; Pezizomycotina; Sordariomycetes; Hypocreomycetidae; Hypocreales; Nectriaceae; Fusarium; Fusarium solani species complex |
| **otu3159** | Eukaryota; Fungi; Dikarya; Ascomycota; Pezizomycotina; Dothideomycetes; Pleosporomycetidae; Pleosporales; Pleosporineae; Didymellaceae; Phoma |
| **otu3159** | Eukaryota; Fungi; Dikarya; Ascomycota; Pezizomycotina; Dothideomycetes; Pleosporomycetidae; Pleosporales; Pleosporineae; Didymellaceae; Didymella |
| **otu3159** | Eukaryota; Fungi |
| **otu3227** | Eukaryota; Metazoa; Chordata; Craniata; Vertebrata; Euteleostomi; Actinopterygii; Neopterygii; Teleostei; Neoteleostei; Acanthomorphata; Holocentriformes; Holocentridae; Myripristis |
| **otu3412** | Eukaryota; Metazoa; Chordata; Craniata; Vertebrata; Euteleostomi; Actinopterygii; Neopterygii; Teleostei; Neoteleostei; Acanthomorphata; Zeiogadaria; Gadariae; Gadiformes; Gadoidei; Gadidae; Gadus |
| **otu3803** | Eukaryota; Metazoa; Chordata; Craniata; Vertebrata; Euteleostomi; Actinopterygii; Neopterygii; Teleostei; Neoteleostei; Acanthomorphata; Zeiogadaria; Gadariae; Gadiformes; Gadoidei; Gadidae; Gadus |
| **otu3806** | Eukaryota; Metazoa; Chordata; Craniata; Vertebrata; Euteleostomi; Actinopterygii; Neopterygii; Teleostei; Neoteleostei; Acanthomorphata; Zeiogadaria; Gadariae; Gadiformes; Gadoidei; Gadidae; Gadus |
| **otu3806** | Eukaryota; Metazoa; Chordata; Craniata; Vertebrata; Euteleostomi; Actinopterygii; Neopterygii; Teleostei; Neoteleostei; Acanthomorphata; Ovalentaria; Pomacentridae; Acanthochromis |
| **otu3806** | Eukaryota; Metazoa; Chordata; Craniata; Vertebrata; Euteleostomi; Actinopterygii; Neopterygii; Teleostei; Neoteleostei; Acanthomorphata; Ovalentaria; Blenniimorphae; Blenniiformes; Blennioidei; Blenniidae; Salariinae; Salarias |
| **otu4365** | Eukaryota; Metazoa; Chordata; Craniata; Vertebrata; Euteleostomi; Actinopterygii; Neopterygii; Teleostei; Neoteleostei; Acanthomorphata; Zeiogadaria; Gadariae; Gadiformes; Gadoidei; Gadidae; Gadus |
| **otu4369** | Eukaryota; Metazoa; Chordata; Craniata; Vertebrata; Euteleostomi; Actinopterygii; Neopterygii; Teleostei; Neoteleostei; Acanthomorphata; Zeiogadaria; Gadariae; Gadiformes; Gadoidei; Gadidae; Gadus |
| **otu5643** | no BLAST matches |
| **otu7224** | Eukaryota; Metazoa; Chordata; Craniata; Vertebrata; Euteleostomi; Actinopterygii; Neopterygii; Teleostei; Neoteleostei; Acanthomorphata; Zeiogadaria; Gadariae; Gadiformes; Gadoidei; Gadidae; Gadus |
| **otu567** | Eukaryota; Metazoa; Chordata; Craniata; Vertebrata; Euteleostomi; Actinopterygii; Neopterygii; Teleostei; Neoteleostei; Acanthomorphata; Zeiogadaria; Gadariae; Gadiformes; Gadoidei; Gadidae; Gadus |
| **otu1841** | unclassified sequences; environmental samples |
| **otu1841** | Archaea; Euryarchaeota; environmental samples |
| **otu1841** | Archaea; environmental samples |
| **otu2033** | Eukaryota; Viridiplantae; Chlorophyta; Mamiellophyceae; Mamiellales; Bathycoccaceae; Bathycoccus |
| **otu2033** | Bacteria; environmental samples |
| **otu2033** | Bacteria; Pseudomonadota; Gammaproteobacteria; Pseudomonadales; Pseudomonadaceae; Pseudomonas; environmental samples |
| **otu2033** | Bacteria; Pseudomonadota; Alphaproteobacteria; Rickettsiales; Anaplasmataceae; Anaplasma |
| **otu2568** | Eukaryota; Metazoa; Chordata; Craniata; Vertebrata; Euteleostomi; Actinopterygii; Neopterygii; Teleostei; Neoteleostei; Acanthomorphata; Zeiogadaria; Gadariae; Gadiformes; Gadoidei; Gadidae; Gadus |
| **otu3349** | Eukaryota; Metazoa; Chordata; Craniata; Vertebrata; Euteleostomi; Actinopterygii; Neopterygii; Teleostei; Neoteleostei; Acanthomorphata; Zeiogadaria; Gadariae; Gadiformes; Gadoidei; Gadidae; Gadus |
| **otu4489** | no BLAST matches_check up |
| **otu4831** | Eukaryota; Metazoa; Chordata; Craniata; Vertebrata; Euteleostomi; Actinopterygii; Neopterygii; Teleostei; Neoteleostei; Acanthomorphata; Zeiogadaria; Gadariae; Gadiformes; Gadoidei; Gadidae; Gadus |
| **otu4831** | Eukaryota; Metazoa; Chordata; Craniata; Vertebrata; Euteleostomi; Actinopterygii; Neopterygii; Teleostei; Neoteleostei; Acanthomorphata; Eupercaria; Spariformes; Sparidae; Acanthopagrus |

| | |
|---|---|
| **otu1295** | no BLAST matches |
| **otu3413** | no BLAST matches |
| **otu4047** | no BLAST matches |
| **otu365** | Eukaryota; Discoba; Jakobida; Histionina; Jakobidae; Jakoba |
| **otu365** | Bacteria; environmental samples |
| **otu2254** | Eukaryota; Discoba; Jakobida; Histionina; Seculamonas |
| **otu3237** | Eukaryota; Amoebozoa; Discosea; Longamoebia; Centramoebida; Acanthamoebidae; Acanthamoeba |
| **otu6048** | Bacteria; environmental samples |
| **otu6048** | Eukaryota; Rhodophyta; Florideophyceae; Nemaliophycidae; Batrachospermales; Lemaneaceae; Paralemanea |
| **otu6048** | unclassified sequences; environmental samples |

**Supplementary Table 3:** Community characteristics in Dataset 1 and 2. The outlier sequences identified did not make up much of the population pool. The misclassified outlier sequences did not contribute significantly to the population pool on a sample basis and generally contributed little to the overall relative abundance in each sample.

| | n outliers | % of population pool | Max ASV sample loss | Relative abundance per sample (± SD) | Max sample abundance |
|---|---|---|---|---|---|
| Dataset 1 | 37 | 1.2% | 8 | Water: 0.1% (±0.06%)<br>Gut: 0.7% (±1.5%) | 9.0% |
| Dataset 2 | 42 | 1.3% | 5 | Water&feed: 0.04% (±0.1%)<br>Larvea:0.06% (±0.24%) | 2.4% |

**Supplementary Table 4:** The average±SD similarity using different similarity indices comparing each samples composition between the full and outlier-free dataset.

| | BC | Sørensen | uw UniFrac | w UniFrac | n uw UniFrac < 0.95 |
|---|---|---|---|---|---|
| Dataset 1 | 1.00±0.01 | 0.99±0.03 | 0.37±0.22 | 0.97±0.07 | 82 of 88 |
| Dataset 2 | 1.00±0.001 | 1.00±0.004 | 0.97±0.044 | 1.00±0.001 | 51 of 197 |

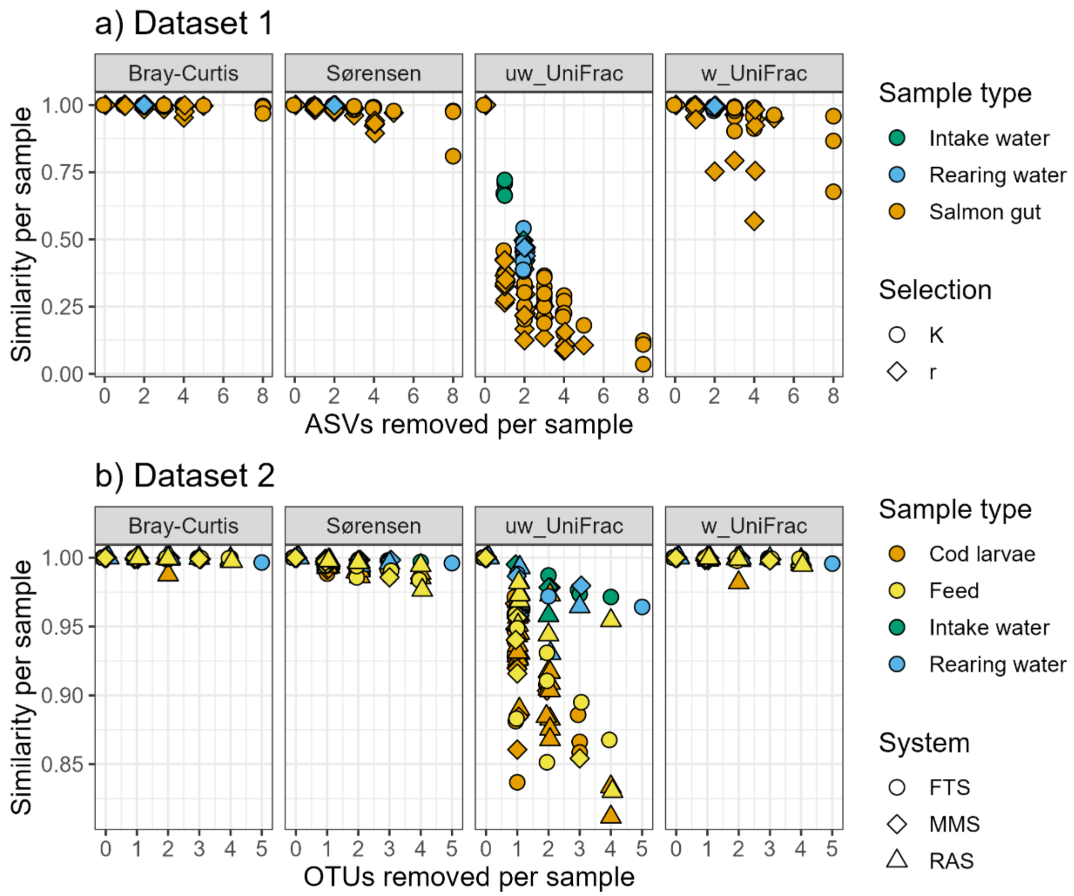


**Supplementary Figure 3:** The Bray-Curtis, Sørensen, unweighted UniFrac and weighted UniFrac similarity for each sample between the datasets with and without the misclassified outliers in a) Dataset 1 and b) Dataset 2.

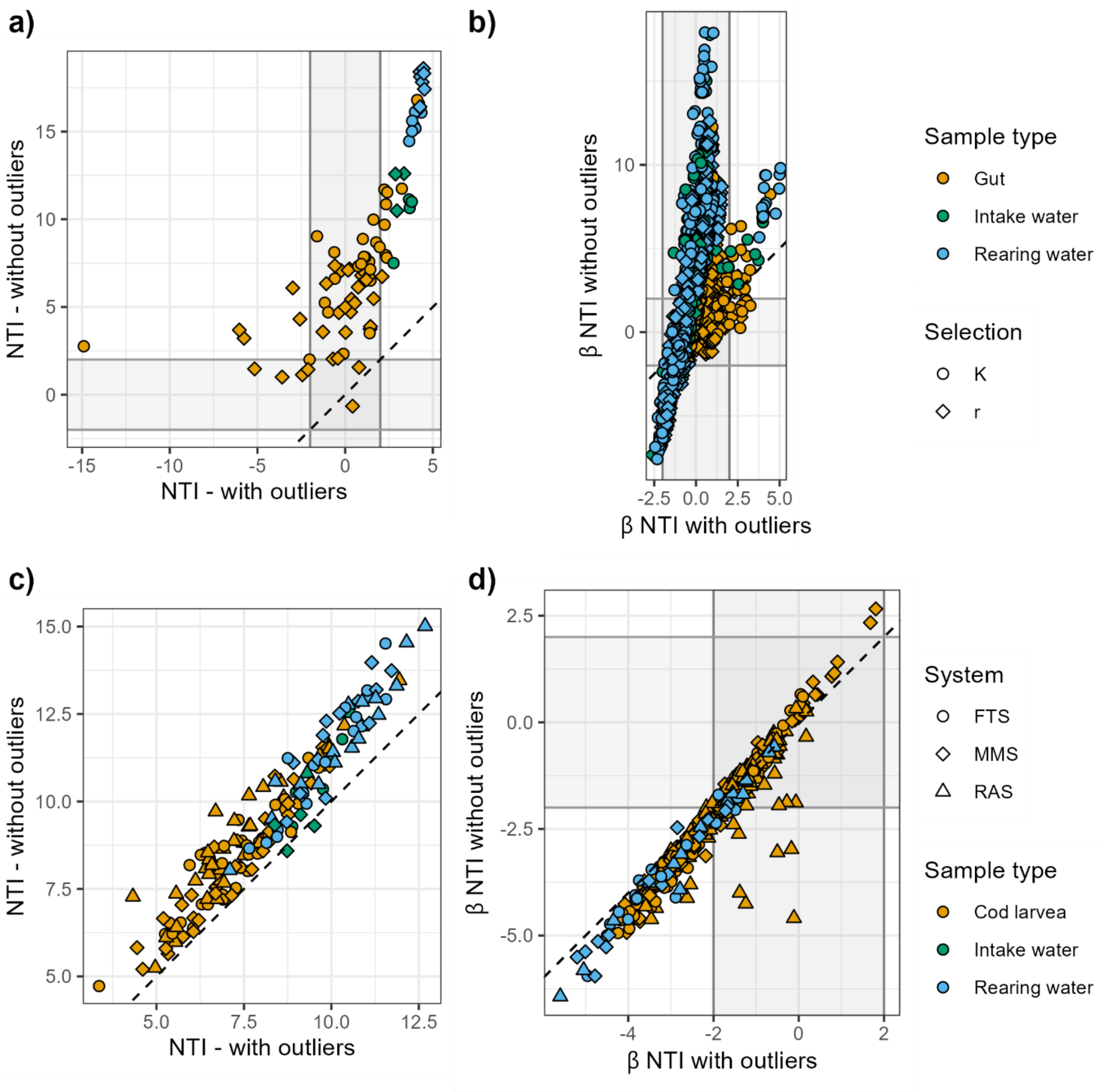


**Supplementary Figure 4:** Differences in estimated NTI and βNTI from the data with and without outlier sequences. Difference in NTI for each sample in a) Dataset 1 (salmon alevin) and c) Dataset 2 (cod larvae). Differences in estimated βNTI between pairs of samples in b) Dataset 1 and d) Dataset 2. Only sample comparisons within the same sample type and sampling water selection regime are shown. The dashed line shows the 1:1 relationship. Values within the grey area are within two standard deviations of the null model.

**Supplementary Table 5:** Overview of how the removing the outlier sequences affected the number of ASVs or OTUs in the dataset and the estimated NTI and βNTI (mean±standard deviation) for the gut and water samples.

| | | **Full dataset** | **Outlier-free dataset** |
|---|---|---|---|
| **Dataset 1** | n ASVs | 3186 | 3149 |
| | NTI Salmon gut samples | -0.04 ±2.74 | 6.07±3.12 |
| | NTI rearing water | 4.14±0.27 | 16.61±1.34 |
| | NTI intake water | 3.31±0.52 | 11.54±2.52 |
| | βNTI Salmon gut samples | 0.15±0.48 | 2.10±1.93 |
| | βNTI rearing water samples | -1.92±0.53 | -5.37±1.08 |
| | βNTI intake water samples | -1.10±0.68 | -1.16±2.52 |
| **Dataset 2** | n OTUs | 3336 | 3294 |
| | NTI Cod larvae samples | 7.23±1.49 | 8.43±1.70 |
| | NTI rearing water samples | 10.04±1.26 | 11.59±1.66 |
| | NTI intake water samples | 9.39±0.92 | 10.29±1.33 |
| | βNTI Cod larvae samples | -2.00±1.07 | -2.24±1.28 |
| | βNTI rearing water samples | -3.08±1.35 | -3.45±1.52 |

**Supplementary Table 6:** Overview of how the metacommunity richness affected the number of ASVs or OTUs in the metacommunity and the estimated NTI and βNTI (mean±standard deviation) for the gut and rearing water samples

| | **Metacommunity** | **All samples** | **Rearing+Gut** | **Gut** |
|---|---|---|---|---|
| **Dataset 1** | n ASVs | 3149 | 2509 | 2106 |
| | NTI Salmon gut samples | 6.07 ±3.11 | 5.16±2.84 | 5.49±2.97 |
| | NTI rearing water | 16.61±1.34 | 14.27±0.86 | - |
| | βNTI Salmon gut samples | 2.10±1.93 | 2.55±2.02 | 2.41±1.99 |
| | βNTI rearing samples | -5.37±1.08 | -4.41±1.05 | - |
| **Dataset 2** | n OTUs | 3294 | 3064 | 1883 |
| | NTI Cod larvae samples | 8.43±1.70 | 8.15±1.63 | 7.13±1.31 |
| | NTI rearing water | 11.59±1.66 | 11.08±1.54 | - |
| | βNTI Cod larvae gut samples | -2.24±1.28 | -2.15±1.28 | -1.61±1.21 |
| | βNTI rearing samples | -3.45±1.52 | -3.31±1.46 | - |

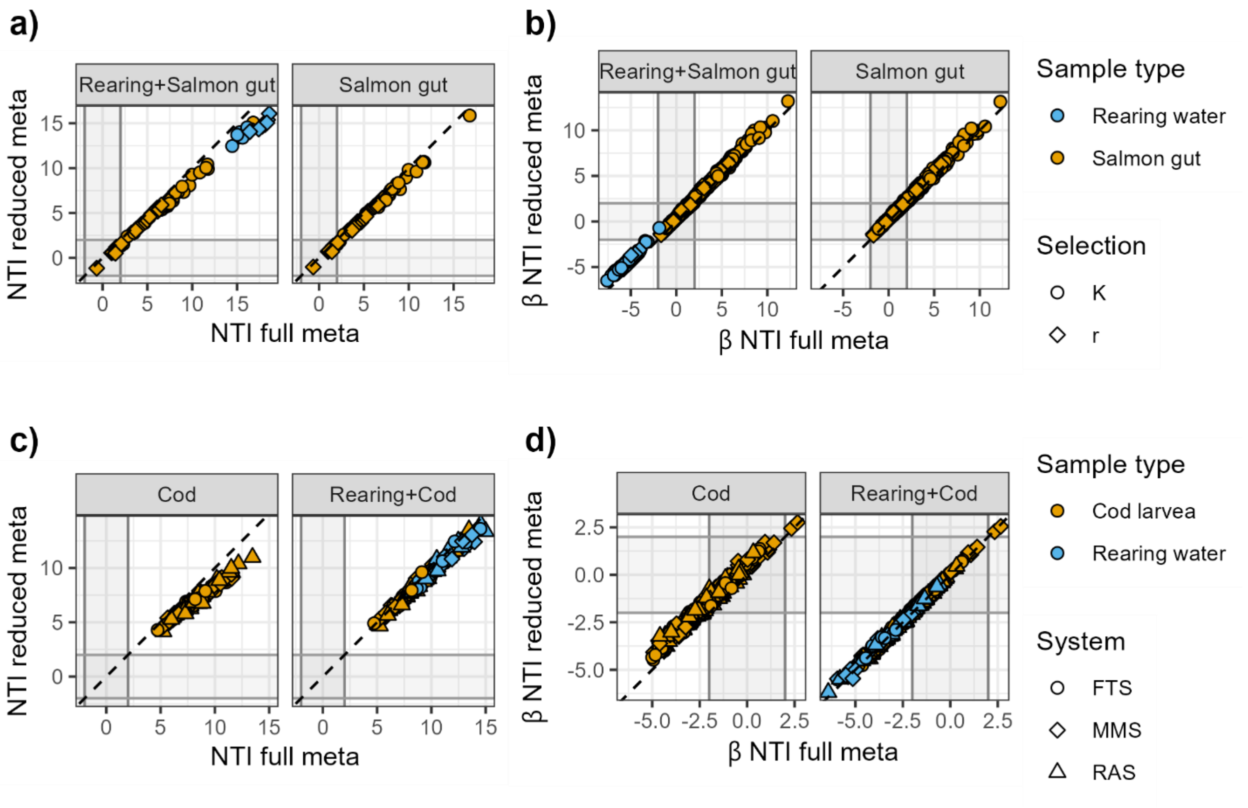


**Supplementary Figure 5:** The NTI and βNTI estimated in the full and reduced metacommunities. Comparison of NTI in the reduced and full metacommunity in a) Dataset 1 and c) Dataset 2. Comparison of βNTI in the reduced and full metacommunity in b) Dataset 1 and d) Dataset 2. The dashed line indicates a 1:1 ratio. Values within the grey areas are within 2 standard deviations of the null model. Only sample comparisons within the same sample type, sampling day and water selection regime/system are shown.
